# Influenza vaccination in the elderly: 25 years follow-up of a randomized controlled trial. No impact on long-term mortality

**DOI:** 10.1371/journal.pone.0216983

**Published:** 2019-05-23

**Authors:** Ruud Andreas Fritz Verhees, Carel Thijs, Ton Ambergen, Geert Jan Dinant, Johannes Andreas Knottnerus

**Affiliations:** 1 Department of Family Medicine, School for Public Health and Primary Care (CAPHRI), Maastricht University, Maastricht, The Netherlands; 2 Department of Epidemiology, School for Public Health and Primary Care (CAPHRI), Maastricht University, Maastricht, The Netherlands; 3 Department of Methodology and Statistics, Maastricht University, Maastricht, The Netherlands; University of British Columbia, CANADA

## Abstract

Influenza vaccination is proven effective in preventing influenza. However, long-term effects on mortality have never been supported by direct evidence. In this study we assessed the long-term outcome of influenza vaccination on mortality in the elderly by conducting a 25-year follow-up study of a RCT on the efficacy of influenza vaccination as baseline. The RCT had been conducted in the Netherlands 5 years before vaccination was recommended for those aged >65 and 17 years before recommending it for those aged >60. The RCT included 1838 community-dwelling elderly aged ≥ 60 that had received an intramuscular injection with the inactivated quadrivalent influenza vaccine (n = 927) or placebo (n = 911) during the 1991/1992 winter. In our follow-up study, outcomes included all-cause mortality, influenza-related mortality and seasonal mortality. Unadjusted and adjusted hazard ratios (HRs) were estimated by Cox regression and sub-hazard ratios (SHRs) by competing risk models. Secondary analyses included subgroup analyses by age and disease status. The vital status up to January 1, 2017 was provided in 1800/1838 (98%) of the cases. Single influenza vaccination did not reduce all-cause mortality when compared to placebo (adjusted HR 0.95, 95% CI 0.85−1.05). Also, no differences between vaccination and placebo group were shown for underlying causes of death or seasonal mortality. In those aged 60–64, median survival increased with 20.1 months (95% CI 2.4–37.9), although no effects on all-cause mortality (adjusted HR 0.86, 95% CI 0.72−1.03) could be demonstrated in survival analysis. In conclusion, this study did not demonstrate a statistically significant effect following single influenza vaccination on long-term mortality in community-dwelling elderly in general. We propose researchers designing future studies on influenza vaccination in the elderly to fit these studies for longer-term follow-up, and suggest age-group comparisons in observational research.

**Clinical trial registry number:** NTR6179.

## Introduction

Influenza has a major impact on morbidity and mortality, especially among the elderly. In the United States, influenza accounts for about 32 000 (5%) of 600 000 annual senior winter deaths [1,2] and an economic burden of roughly 56 billion US dollars every year [3]. Up to present, vaccination is the most effective method to prevent influenza infection [4]. In our double blind randomized controlled trial (RCT), vaccination was shown effective in preventing influenza in the elderly [5,6]. However, there is a lack of direct evidence on its effectiveness on seasonal and long-term mortality [7].

Worldwide, only three RCTs have evaluated the efficacy of inactivated influenza vaccines on clinical influenza in community-dwelling elderly [5,8,9]. None of these trials had the primary objective or power to assess mortality. Hence, current knowledge on mortality is based on observational research. Multiple recent meta-analyses of these studies concluded that vaccination can reduce mortality by 30 to 50 percent [10–12]. However, observational studies are susceptible for selection bias and confounding [13–15]. Therefore, various attempts have been made to reduce bias, for instance by adjusting for confounders [16,17], adjusting analyses [18,19], and using advanced study designs [20]. Despite this, critics state that the role of vaccination in the elderly is unclear and an adequately powered, placebo-controlled trial should be undertaken [21,22]. However, such a trial is unlikely to gain ethical approval [7,13,23]. Thus, conclusions on the effectiveness of influenza vaccination on mortality in community-dwelling elderly may remain based on observational studies and the inference that reduction of influenza implies reduction of influenza-related complications including mortality.

Besides the effects on short-term (i.e. seasonal or annual) mortality, the long-term potential of vaccination has never been evaluated in experimental settings. Studies have suggested that vaccination might prevent influenza-related complications such as cardiovascular events [24,25], reduce severity of community-acquired pneumonias [26], and reduce the risk of hospitalization [27,28]. It has also been suggested that influenza vaccination might provide residual protection against influenza strains that occur later in life [29], and elicit immune memory [30,31]. Hence, we hypothesize that by preventing (accumulation of) influenza-related complications and residual protection that lasts more than just one influenza season, influenza vaccination could reduce mortality on the longer term. Considering the age-associated decline of the immune system and the large group of influenza vaccination-naïve patients by the time of conducting the trial, describing long-term outcome of influenza vaccination might be particularly interesting in the younger elderly.

Evaluating the long-term outcome of vaccination is relevant since it contributes to the evidence of the effects on mortality from a long-term perspective and might support patient and policy decision making. Therefore, we have used our 1991 trial as baseline for a long-term follow-up study to explore the long-term outcome of influenza vaccination on mortality and underlying causes of death in community-dwelling elderly.

## Materials and methods

We conducted a 25-year follow-up on mortality of a RCT on the efficacy of influenza vaccination in the elderly in the 1991/92 winter season.

### Study population

The 1991 trial involved 34 family physicians in 15 practices in the Netherlands. In total, 1838 patients aged >60 years, not known to belong to those high-risk groups in which vaccination was recommended, were included. At that time, age was no criterion for recommending vaccination. Cardiovascular, pulmonary or metabolic problems that did not require vaccination according to the family physician were reported in 490 participants. Following randomization, patients received an intramuscular injection in the deltoid area with the inactivated quadrivalent influenza vaccine (n = 927) or with saline solution (n = 911). We refer to our original publication for more detailed information on the trial intervention [5].

### Data collection

**Fig 1:**
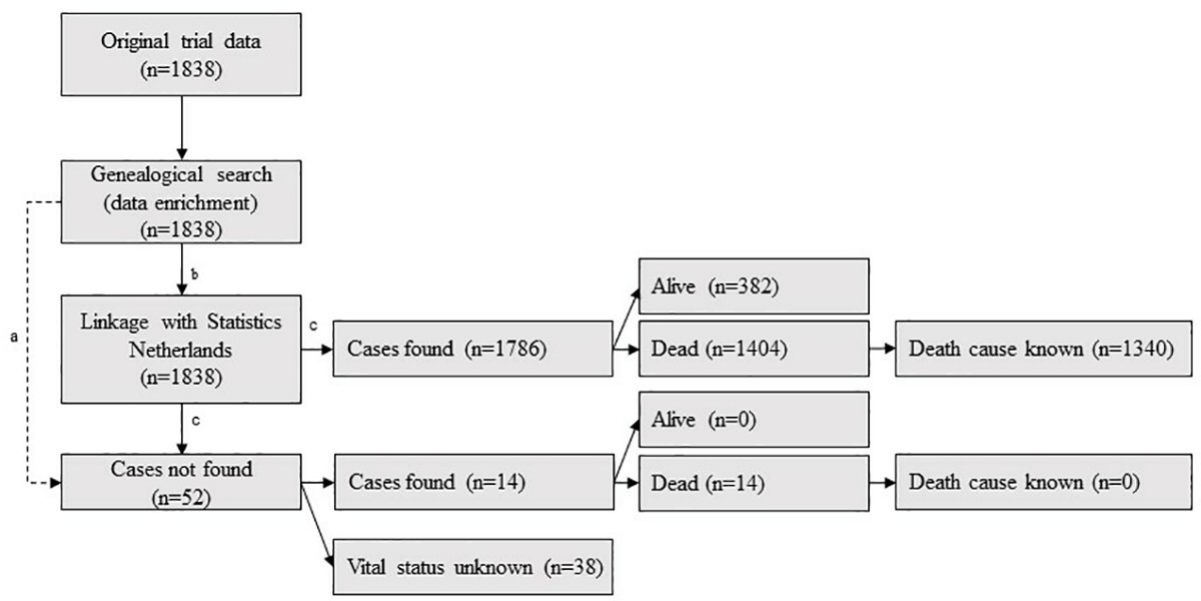
Process of data collection for obtaining mortality statistics. a If cases were not found by the CBS, data retrieved by the genealogical search was used in order to obtain follow-up information. b Genealogical search was used for maximizing CBS-search results. c Life status as known by 1 January, 2017.

We completed the 1991 trial data with mortality statistics. In the Netherlands, Statistics Netherlands (Centraal Bureau voor de Statistiek, CBS), keeps person records including information on the vital status and death certificates. Person records are linked to a unique identity number. To maximize the yield of the CBS search, we carried out an extensive genealogical search retrieving all relevant information useful for tracking identity numbers (S1 Text, S1 and S2 Figs). A summary of the search is given in Fig 1.

If data retrieved by the genealogic search conflicted with patient characteristics as reported in the 1991 trial (e.g. differences in age or sex), the conflicting cases were checked for clerical errors in the database by looking up the informed consent forms of the 1991 trial. If no clerical errors had been made and the genealogic search yielded one official person record only, then the patient characteristics were updated using the new information found.

We defined six mortality endpoints based on the diagnostic codes of the *International Classification of Diseases*, *Ninth Revision* (*ICD-9*) for deaths prior to 1996 and *ICD-10* for deaths that occurred in 1996 or later [32,33]: all-cause deaths (all ICD codes), underlying respiratory deaths (ICD-9 codes 460−519, ICD-10 codes J00−J99), underlying circulatory deaths (ICD-9 390−459, ICD-10 codes I00−I99), underlying respiratory or circulatory deaths, and underlying pneumonia or influenza deaths (ICD-9 codes 480−487, ICD-10 codes J09−J18). To ensure that influenza and pulmonary antecedents possibly related to influenza were noted, we modelled the sixth category (“pulmonary related mortality”); taking into account any pulmonary condition registered as antecedent or primary cause of death. All-cause mortality, primary and antecedent death causes were available up to 1 January, 2017; 1 January, 2016; and 1 January, 2013, respectively. For each endpoint, seasonal mortality was assessed. Seasonal mortality was defined as any death occurring between December up to June, assuming that influenza-related mortality is likely to occur during or shortly after influenza epidemics, which in the Netherlands can take up until the end of April [34].

### Bias

Selection bias and confounding by indication at baseline were avoided since this study is based on double blind placebo-controlled randomization [5]. If genealogical search data conflicted with personal records registered in the 1991 trial, a four member panel blinded for the trial intervention updated the personal records only after agreeing on data reliability. The randomization was maintained for all subjects, ensuring intention-to-treat analysis.

### Variables

Eight baseline independent variables were considered, including: trial intervention (trial vaccination or placebo), age at trial intervention, sex, disease status as known in 1991, smoking status and previous vaccination status (influenza vaccination in 1989/1990 and 1990/1991). In the 1991 trial, participants with multiple comorbidities had been assigned to one single disease group only (e.g. a patient with coronary sclerosis and chronic obstructive pulmonary disease was assigned to disease group “cardiovascular disease”). In order to optimize data usage in our follow-up study, we choose to reclassify the complete disease status as known in 1991 into each of the underlying disease clusters that had been registered; i.e. cardiovascular disease, pulmonary disease and diabetes mellitus. Thus, a patient with coronary sclerosis and chronic obstructive pulmonary disease was now assigned to both “cardiovascular disease” and “pulmonary disease”.

### Statistical analysis

Kaplan-Meier survival curves were plotted to illustrate the potential association between trial vaccination and survival. We performed univariate and multivariable Cox proportional hazards regression analyses to estimate the hazard ratio (HR) and 95% confidence intervals for all-cause mortality by trial vaccination. Covariates were added as dichotomous variables (trial intervention, sex, cardiovascular disease, pulmonary disease, diabetes mellitus, previous vaccination status), ordinal (smoking status) or continuous variable (age at trial intervention). We used all covariates and all possible interaction terms in the multivariable Cox regression analyses, dropping the least significant terms by backward stepwise approach (P > 0.05). The proportional hazard assumption was checked comparing estimated ln(-ln) survivor curves. To evaluate the relation between trial vaccination and seasonal or specific causes of death by means of sub-hazard ratios (SHRs), we used competing risk regression analysis [35], retaining those covariates and interaction terms with trial intervention (vaccine or placebo) in the model that appeared significant, unless stated otherwise. Since the numbers of events in competing risk regression analyses would be small, only the following additional interactions were assessed: age*sex, age*smoking status, age*cardiovascular disease, age*pulmonary disease, smoking status*pulmonary disease, smoking status*heart disease, smoking status*diabetes mellitus and pulmonary disease*heart disease. Statistical testing was based on two-sided testing with the use of a 5% significance level in all analyses. Subgroups based on age and medical conditions were analyzed for overall mortality only. Underlying causes of death were not analyzed as outcome since numbers of subgroups would be too small and multiple testing could induce false positive findings. Data were missing for smoking status (n = 82) and previous vaccination in 1990 (n = 2). We analyzed our data by assigning the cases with missing smoking status to a separate group and excluding the latter two cases. In order to translate the potential survival benefit to clinical practice, we calculated the difference in median survival between groups. Since this approach was not recorded in our protocol beforehand, this will be considered of additive value. We used IBM SPSS version 23 for Cox regression analysis. Competing risk analyses were performed using competing risk package (cmprsk) in R statistical software, version 3.3.1 (R Foundation for Statistical Computing).

If the CBS-search did not yield information on the vital status of a participant, we used alternative data sources (see result section) to retain a follow-up date and censored the case from that point onwards. To determine whether censoring before end of follow-up posed a potential threat to validity, we carried out sensitivity analyses applying two extreme scenarios; either all participants without actual vital status on 1 January, 2017 died after censoring, or lived up to 1 January, 2017.

Our protocol was registered in the International Clinical Trials Registry Platform (ICTRP) under NTR6179, before data-analysis was performed.

### Ethical considerations

The 1991 trial participants had not been explicitly requested to approve for obtaining information on their vital status later on, since in 1991 this was beyond the scope of the trial. Concerning our follow-up study, the medical ethics committee of the Maastricht University Medical Center concluded this study was not subject to the Medical Research Involving Human Subjects Act. In addition, Statistics Netherlands approved anonymous processing of the mortality data.

As explained in more detail in our supplement (S1 Text), multiple institutional databases have been searched by institute officials: Statistics Netherlands, Municipal Personal Records Database (Gemeentelijke Basisadministratie, GBA), Netherlands Centre for Family History (Centraal Bureau voor Genealogie, CBG) and Regional Historical Centre of Limburg (Rijksarchief Limburg). The aim of the study was explained to each institutional board and/or official. The institutes decided whether mortality dates of the specific cases we requested for could legally be provided. Only after this decision, the databases were searched by the institutes’ officials. No interviews of family members of the deceased participants have been performed in this study. In case former participants were not found in the official databases of the various institutions, the general practitioner was requested to provide this information on the life status (not the cause of death). The general practitioner and his/her staff were motivated to contribute to this study and went through patient records to retrieve information on the exact date of birth. In case of uncertainty, the general practitioners’ staff called a family member of the deceased participant to inform about the date of death (not the death cause). The mortality data were accessed in a fully anonymized way. When mortality dates had been collected, the final step was obtaining the specific death cause of the participants by requesting Statistics Netherlands. Official permission to use these data was granted by the Committee of Statistics Netherlands (report available on request). Information on date of death was important for the CBS in order to search for personalized (and fully anonymized) identification numbers (unique codes corresponding to an unique person). These identification numbers where then used to obtain specific death causes, thus making it impossible to link a specific person to a specific death cause. Our analyses on death causes could only be performed on the online and secured server of Statistics Netherlands, thus ensuring patient privacy. Enriched data (containing information on death causes) could not be exported.

## Results

### Follow-up data

The process of data collection is summarized in Fig 1. During 29 867 person-years of follow-up (median 17.34 years, 95% CI 16.67–18.01), 1418/1838 (77.1%) subjects died. Information on cause of death was retrieved in 1340/1418 (94.5%) cases. A total of 420 cases were censored of which 382 were alive at end of follow-up (January 1, 2017). Thus information on the known vital status was collected in 1800/1838 (97.9%) cases. The remaining 38 cases with unknown vital status were divided equally between vaccine (n = 16) and placebo group (n = 22). Local municipalities provided a follow-up date (e.g. date of rehousing / migration) in 16 cases. In the 22 remaining cases, the last date recorded during the 1991 trial was used to set the censor date.

### Characteristics of study population

Patient characteristics of the 1991 trial and the updated trial are shown in Table 1. Updating the trial data (see “methods”) did not result in any relevant differences between the intervention and placebo group.

**Table 1:** Patient baseline characteristics of the trial population versus the updated trial population.

	Vaccine group (n = 927) No.(%)		Placebo group (n = 911) No.(%)	
Variable	1991 trial	updated trial	1991 trial	updated trial
Sex^a^ Male Female				
	420 (45.3)	413 (44.6)	449 (49.3)	445 (48.8)
	507 (54.7)	514 (55.4)	462 (50.7)	466 (51.2)
Age^a^ 60–64 65–69 70+				
	368 (39.7)	379 (40.9)	396 (43.5)	412 (45.2)
	281 (30.3)	274 (29.6)	249 (27.3)	240 (26.3)
	278 (30.0)	274 (29.6)	266 (29.2)	259 (28.4)
Smoking status (1991) Never Stopped Actual Unknown				
	367(39.6)	367(39.6)	339 (37.2)	339 (37.2)
	312 (33.7)	312 (33.7)	311 (34.1)	311 (34.1)
	206 (22.2)	206 (22.2)	221 (24.3)	221 (24.3)
	42 (4.5)	42 (4.5)	40 (4.4)	40 (4.4)
Pulmonary disease (1991)^b^ Yes No				
	105 (11.3)	129 (13.9)	95 (10.4)	115 (12.6)
		798 (86.1)		796 (87.4)
Heart disease (1991) Yes No				
	125 (13.5)	125 (13.5)	124 (13.6)	124 (13.6)
		802 (86.5)		787 (86.4)
Diabetes Mellitus (1991)^b^ Yes No				
	21 (2.3)	27 (2.9)	20 (2.2)	32 (3.5)
		900 (97.1)		879 (96.5)
Previous vaccination (1989 and/or 1990)^b^ Yes No Unknown				
			
	118 (12.7)	119 (12.9)	120 (13.2)	119 (13.1)
	809 (87.3)	807 (87.1)	791 (86.8)	791 (86.9)
		1(0.1)		1 (0.1)

^a^ Differences in patient characteristics between 1991 trial and updated trial due to either clerical errors made in the original informed consent forms or incorrect data entry at time of trial registration.

^b^ Differences in patient characteristics between 1991 trial and updated trial due to reclassification of covariates.

### Influence of influenza vaccination on mortality and specific causes of death

**Fig 2:**
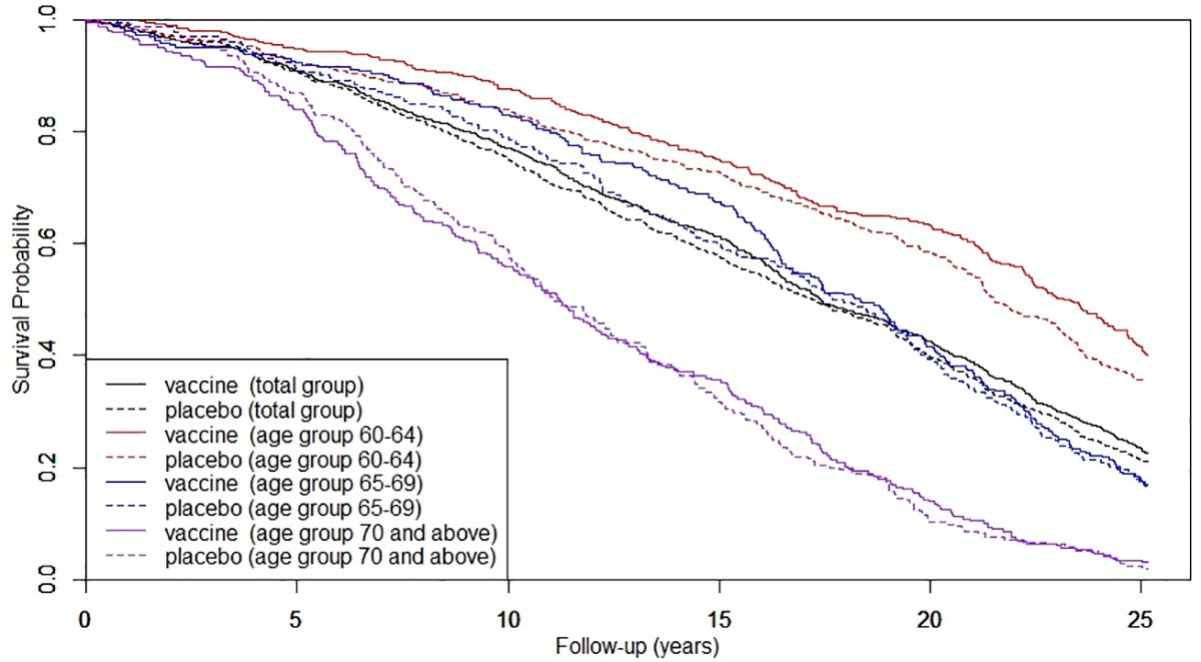
Kaplan-Meier plots for vaccine versus placebo group for different age groups (numbers at risk presented in Table 2).

Median survival for vaccine group was 17.39 years (interquartile range 10.61–24.58) versus 17.20 years for placebo group (10.02–23.70); a difference of 2.2 months (95% CI −14.7 to 19.1). Kaplan-Meier survival plots illustrating survival of different age groups are shown in Fig 2. Corresponding numbers at risk and hazard ratios are listed in Table 2. After adjusting for covariates and taking potential effect modifiers into account, this study found no overall effect of influenza vaccination on mortality (adjusted HR 0.95, 95% CI 0.85–1.05).Sensitivity analyses (not presented) showed consistent results.

**Table 2:** Numbers at risk and hazard ratios corresponding to Kaplan-Meier plots for vaccinated and unvaccinated participants of the 1991 trial.

Age group	vaccine / placebo	Follow-up (years)						Unadjusted HR (95% CI)	P Value
		0	5	10	15	20	25		
All ages	Vaccine	927	835	708	563	389	212	0.94 (0.85–1.05)	0.29
	Placebo	911	812	677	518	357	187		
60–64	Vaccine	379	359	332	284	239	155	0.86 (0.72–1.03)	0.10
	Placebo	412	376	342	297	239	142		
65–69	Vaccine	274	252	227	184	113	48	0.95 (0.79–1.15)	0.61
	Placebo	240	214	185	140	92	40		
70+	Vaccine	274	224	149	95	37	9	0.97 (0.81–1.15)	0.72
	Placebo	259	222	150	81	26	5		

HR: hazard ratio.

Overall hazard ratios contrasting trial vaccination versus placebo for different outcome measures are presented in Table 3. Neither overall nor seasonal differences between trial vaccination and placebo on all-cause mortality or any underlying cause of death were shown. The interaction term between trial vaccination and diabetes mellitus was significant (P = 0.02) for all-cause mortality. Also interaction terms between trial vaccination and pulmonary disease (P = 0.04) and trial vaccination and smoking (P = 0.04) were significant for pulmonary related seasonal mortality. Subsequent analysis for participants with and without pulmonary disease for this outcome demonstrated sub-hazard ratios (SHRs) of 0.56 (95% CI 0.31–1.01) and 1.21 (95% CI 0.83–1.77) respectively (S3 and S4 Figs). The interaction term between trial vaccination and smoking status was not retained in the model since there were no important differences in the SHRs for the model with and without interaction for each of the smoking categories. The SHR for all-cause mortality stratified by diabetes mellitus is shown in Table 4 and did not demonstrate a statistically significant effect of trial vaccination after adjusting the model to the small group size (n = 59).

**Table 3:** Adjusted and unadjusted hazard ratio (HR) and sub-hazard ratio (SHR) of trial vaccination for all-cause mortality and underlying cause of death respectively.

Outcome	Vaccine group (n = 927)		Placebo group (n = 911)			
	No.(%)	rate	No.(%)	rate	Unadjusted (S)HR (95% CI)	Adjusted (S)HR (95% CI)^a^
All-cause mortality Full year Seasonal^b^						
	711(76.7)	4.65	707(77.6)	4.85	0.94 (0.85–1.05)	0.95 (0.85–1.05)^c^^,^^d^
	378(40.8)	2.47	381(41.8)	2.61	0.95 (0.83–1.10)	0.94 (0.81–1.08)
Pulmonary or circulatory deaths Full year Seasonal^b^						
					
	329(35.5)	2.18	324(35.6)	2.25	0.99 (0.85–1.15)	0.96(0.82–1.13)
	185(20.0)	1.23	188(20.6)	1.31	0.95 (0.78–1.17)	0.93(0.75–1.14)
Pulmonary deaths Full year Seasonal^b^						
	80(8.6)	0.53	75(8.2)	0.52	1.04 (0.76–1.43)	1.04 (0.76–1.44)
	56(6.0)	0.37	50(5.5)	0.35	1.10 (0.75–1.60)	1.06 (0.72–1.56)
Circulatory deaths Full year Seasonal^b^						
	249(26.9)	1.65	249(27.3)	1.73	0.97 (0.81–1.16)	0.96 (0.80–1.15)
	129(13.9)	0.86	138(15.1)	0.96	0.90 (0.71–1.15)	0.90 (0.71–1.15)
Influenza/pneumonia deaths Full year Seasonal^b^						
					
	35(3.8)	0.23	28(3.1)	0.19	1.22 (0.74–2.01)	1.20 (0.73–1.97)
	25(2.7)	0.17	21(2.3)	0.15	1.16 (0.65–2.07)	1.13 (0.63–2.03)^e^
Pulmonary related deaths Full year Seasonal^b^						
	140(15.1)	0.99	134(14.7)	0.99	1.02 (0.81–1.29)	1.07(0.84–1.37)
	81(8.7)	0.57	86(9.4)	0.63	0.92 (0.68–1.24)	0.96(0.70–1.31)^f^

HR:hazard ratio; SHR:sub-hazard ratio.

Percentages are calculated by: n(events)/N(patients). N(patients)_vaccine_ = 927, N(patients)_placebo_ = 911.

^a^ (Sub-) hazard ratios adjusted for age, sex, smoking status, pulmonary disease, heart disease, diabetes mellitus, previous vaccination status and first-order interactions: trial vaccination by all covariates and 8 additional interactions (i.e. age * sex, age * smoking status, age * pulmonary disease, age * heart disease, smoking status * pulmonary disease, smoking status * heart disease, smoking status * diabetes mellitus, pulmonary disease * heart disease), unless otherwise specified. 2 individuals were excluded due to missing data.

^b^ Defined as death occurring annually between the 1^st^ of December and the 31^st^ of May.

^c^ (Sub-) hazard ratio adjusted for all potential first-order interactions, given the large numbers of events (n = 1418).

^d^ Significant interaction term (p = 0.02) between trial vaccination and diabetes mellitus.

^e^ No adjustments made for interactions, given the small numbers of events (n = 46).

^f^ Significant interaction term (p = 0.04) between trial vaccination and pulmonary disease, and between trial vaccination and smoking (p = 0.04).

**Table 4:** Adjusted and unadjusted hazard ratio (HR) for all-cause mortality by subgroups by trial vaccination.

Subgroups	No.events / total(%)		Unadjusted HR (95%CI)	Adjusted HR (95%CI)^a^
	Vaccine group	Placebo group		
Age 60–64 (n = 791)	226/379(59.6)	262/412(63.6)	0.86(0.72–1.03)	0.86 (0.72–1.03)
Age 65–69 (n = 514)	226/274(82.5)	195/240(81.3)	0.95(0.79–1.15)	0.99(0.82–1.20)
Age 70+ (n = 533)	259/274(94.5)	250/259(96.5)	0.97(0.81–1.15)	1.04(0.87–1.23)
Pulmonary disease (n = 244)	112/129(86.8)	101/115(87.8)	0.90(0.69–1.18)	1.13(0.86–1.48)
Cardiovascular disease (n = 249)	107/125(85.6)	110/124(88.7)	0.86(0.66–1.13)	0.88(0.67–1.15)
Diabetes mellitus (n = 59)	26/27(96.3)	28/32(87.5)	1.38 (0.81–2.35)	1.53(0.89–2.62)
Pulmonary and/or cardiovasc. disease and/or DM (n = 490)	218/251(86.9)	209/239(87.4)	0.92(0.76–1.11)	0.97(0.80–1.17)
Previous vaccination (n = 238)	101/119(84.9)	95/119(79.8)	1.04(0.78–1.37)	1.01 (0.76–1.34)

HR: hazard ratio; DM:diabetes mellitus.

Data are n(events)/N(patients).

^a^ Hazard ratios adjusted for sex, age, smoking status, pulmonary disease, heart disease, diabetes mellitus and previous vaccinations (2 individuals were excluded due to missing data).

Subgroup analyses for different health conditions, previous vaccinations and age groups (Table 4), did not show a significant effect of vaccination on survival. Vaccination of the younger elderly (60–64 years) demonstrated the highest reduction in mortality rate; i.e. 14% (adjusted HR 0.86, 95% CI 0.72–1.03). In this age group, median survival time was 23.18 years (3^rd^ quartile 15.00) in the vaccine group versus 21.50 years in placebo group (3^rd^ quartile 13.67), an absolute difference of 20.1 months (95% CI 2.4–37.9). Only the 3^rd^ quartile of this group could be provided since at end of follow-up, over 25% of the population was still alive.

## Discussion

In this study based on a RCT, we could not demonstrate a relation between one single influenza vaccination and mortality or specific causes of death in the elderly after 25 years of follow-up. Also no effect of vaccination on seasonal mortality was shown. Although not statistically significant, survival was in favor of the vaccine group during the entire length of follow-up, with hazard rates being constant over time.

Subgroup analyses suggested that the effects of vaccination on mortality could be more prominent in those aged 60−64 years (adjusted HR 0.86, 95% CI 0.72–1.03). This is supported by literature stating that influenza-specific humoral immunity and clinical effectiveness of influenza vaccination are negatively influenced by age [36–38]. Analyzing the younger elderly as such is relevant given the ongoing debate on the influence of age on vaccination effectiveness. Moreover, influenza-associated mortality and hospitalization are substantial also in this group [39]. Our finding of a difference in median survival, i.e. 20.1 months (95% CI 2.4–37.9), could be considered additional supportive evidence, not as proof of efficacy in this group.

Other subgroup analyses based on pre-existing cardiovascular and/or pulmonary disease or on previous vaccination did not show clear indications for effects of vaccination on all-cause mortality. Although patients with lung disease might be more prone to harmful effects of influenza, we could not demonstrate a clear effect of vaccination on pulmonary-related seasonal mortality in this subgroup (adjusted SHR 0.56, 95% CI 0.31–1.01). We found a sub-hazard ratio for vaccination of 1.53 (95% CI 0.89–2.62) in diabetic patients. However, this analysis is based on low numbers (n = 59) and the survival plots for diabetics showed an unfavorable effect of vaccination just 15 years after trial intervention (S5 Fig).

Despite the biological plausibility that vaccinating the youngest elderly would be more effective, it may seem unlikely that the effect size of trial vaccination on mortality in the younger elderly (HR 0.86, 95% CI 0.72–1.03) is explained by administration of one single vaccination in 1991/1992. However, by that time uptake of influenza vaccinations was low and vaccination recommendations for those aged ≥65 were only introduced in 1996. Thus, especially for those aged 60−64, the 1991 trial vaccination was likely to have been the first and for a long time only influenza vaccination received. Since similar vaccine components as used in the 1991 trial showed to provide protection against some of the influenza strains that circulated the five subsequent years after trial intervention, this might have had implications for the long-term efficacy of the trial vaccination. For instance, it has been shown that influenza vaccines can elicit immune memory and protect against drift variants or non-homologous strains [30,31]. Also, McLean et al. demonstrated that protection against influenza could also be elicited by vaccination during the previous season. This residual protection even occurred despite that the predominant viruses were antigenically distinct from previous season vaccine components [29]. It should be stated that these studies included mainly young(er) individuals and thus might not fully apply to elderly due to their diminished immune responses [40]. Yet eventually, we cannot ignore that in our trial, random allocation to vaccine or placebo group is at the basis of differences in survival between vaccine and placebo group in the younger aged. In addition, subgroup analyses of the age groups 65−69 and 70+ showed hazard ratios near 1.0. Taking these differences and the study power into account, in our view a protective effect of vaccination on mortality in the youngest age group cannot be ruled out.

Methodologically, it is difficult to compare our study results with literature since studies predominantly evaluate seasonal effects of vaccination on the short term. In their RCT, Praditsuwan et al. evaluated mortality after one year. Whereas underpowered for this secondary endpoint, they could not demonstrate a relation between vaccination and mortality. A cohort-study showed that vaccination was associated with lower mortality in community-dwelling elderly during 6 months of follow-up, but did not prevent death in the subgroup of healthy elderly (relative risk 0.87, 95% CI 0.62–1.20) [41]. However, in that study vaccination did reduce mortality in elderly with comorbidity (relative risk 0.67, 95% CI 0.48–0.94). Moreover, a meta-analysis evaluating the effect of (different types of) influenza vaccination in a diverse population (various ages, largely elderly known with cardiovascular comorbidity), showed that influenza vaccination may reduce combined cardiovascular mortality [24]. Our study did not demonstrate such a comorbidity-related subgroup effect, but may be difficult to compare with these studies since medical problems registered in our 1991 trial did not require vaccination according to the physician and thus might have been of minor clinical relevance.

A limitation of our study concerns its power. The 1991 trial was not designed to evaluate short-term mortality and thus numbers are too small to relate vaccination directly to influenza deaths in the influenza season that year. The high rate (97.9%) of complete follow-up did result in high numbers of person years of follow-up and events, thus increasing the study power. However, post-hoc power calculation showed that only hazard ratios ≤ 0.85 could have been proven significant. Therefore, we cannot exclude that our group size was too small to be able to show effects of vaccination on mortality that are relevant at the population level.

It would have been interesting if more information on revaccination after 1991 was available because this may have affected mortality. Since in the Netherlands the legal obligation to store medical files expires after 15 years, reconstruction of these data is problematic. Because participants were not de-blinded at end of the trial, both randomized groups are likely to have been (re)vaccinated in equal proportions. Since differences in vaccination status between vaccination and placebo group will have become smaller over time, it is likely this has diluted the effect of our 1991 trial vaccination.

An important strength of our study concerns the follow-up being based on a well-documented RCT. In this trial the vaccine was well matched with the epidemic influenza strains, possibly maximizing the efficacy of vaccination on morbidity, and–as set out in our hypothesis–on long-term mortality as an outcome. Since our trial was found to be the only one of the three previously mentioned RCTs that could be evaluated on long-term mortality and new placebo-controlled trials are unlikely to be approved by ethical boards, the results from our study might remain the only direct evidence available on long-term effects of influenza vaccination on mortality in community-dwelling elderly.

## Conclusions

In conclusion, this study did not demonstrate an effect of influenza vaccination on long-term mortality in community-dwelling elderly in general. However, taking the limited power of our study into account, the consistent and relevant findings in the subgroup aged 60–64 years merit a long-term study of the effect of vaccination on mortality the elderly. Therefore, we recommend researchers intending to conduct studies on the efficacy of influenza vaccines, to accustom these studies for longer follow-up. Moreover, these results could encourage researchers to compare long-term survival in younger and older vaccinated elderly and to study the long-term effect of influenza vaccination on immune memory.

## Supporting information

S1 Text Method used for genealogical data collection. (DOCX)

S1 Fig Process of data enrichment by genealogical search. (DOCX)

S2 Fig Definite search process conducted by Statistics Netherlands (CBS). (DOCX)

S3 Fig Cumulative incidence plot; pulmonary related seasonal deaths in participants with lung disease. (DOCX)

S4 Fig Cumulative incidence plot; pulmonary related seasonal deaths in participants without lung disease. (DOCX)

S5 Fig Crude Kaplan-Meier survival curves for vaccinated and unvaccinated diabetic patients. (DOCX)
