# Supplementary material for: Influenza vaccination in the elderly: 25 years follow-up of a randomized controlled trial. No impact on long-term mortality
Source: PLoS One. 2019 May 23;14(5):e0216983. doi: 10.1371/journal.pone.0216983 (PMC6532873; doi:10.1371/journal.pone.0216983)
Supplement: S1 Text — (DOCX) [file pone.0216983.s001.docx]

**S1 Text. Method used for genealogical data collection**

An extensive genealogical search was conducted in order to maximize the yield of the search performed by Statistics Netherlands (CBS). This genealogical search is summarized in supplement S1 Fig. Since multiple institutes were involved in the process of data collection, an explanatory box is shown below this text. On our request the Municipal Personal Records Database (Gemeentelijke Basisadministratie, GBA) was searched by a government official in December 2015 to identify whether the participants had died, and provide the date of death if applicable (i.e. the “vital status”). In case of negative findings we contacted the participants’ family practice to enquire about the vital status. If this could not be provided (e.g. due to migration), we requested the Netherlands Centre for Family History (Centraal Bureau voor Genealogie, CBG) and subsequently, an archivist of the Regional Historical Centre of Limburg (Rijksarchief Limburg) to conduct a search in the national and the regional archives. If a search for an individual was not successful, the vital status was checked by the local municipality where the former participant had been living according to our most recent information. Search results of the family practice were double checked by the municipalities. In case patient characteristics retrieved by our genealogical search conflicted with the characteristics as recorded in our database originating from 1991, we checked the original informed consent forms for inconsistencies and adjusted the data based on these findings. Finally, Statistics Netherlands obtained the unique identification numbers based on our genealogic search (S2 Fig.). These numbers were then used to look up the date of death and underlying death cause in the CBS-database.

**Explanatory box. Institutes that collect and preserve mortality data in the Netherlands**

- **The Municipal Personal Records Database (Gemeentelijke Basisadministratie, GBA)** The national database maintained under the authority of the Dutch Ministry of the Interior and Kingdom Relations, that digitally registers official personal records of all Dutch residents and residents who passed away after October 1994.
- **Statistics Netherlands (Centraal Bureau voor de Statistiek, CBS)**

The national autonomous administrative body that is responsible for collecting and processing national data in order to publish official statistics.

- **Regional Historical Centre of Limburg (Rijksarchief Limburg)**

A statutory authority that collects and preserves a wide range of documents relating to the history of Limburg and its people for the purpose of the public and regional municipalities.

- **Netherlands Centre for Family History (Centraal Bureau voor Genealogie, CBG)**

Both an information center and center of expertise, the CBG provides sources, advice and research in the field of genealogy, heraldry, family names and related domains in the Netherlands.
