# Supplementary material for: Influenza vaccination in the elderly: 25 years follow-up of a randomized controlled trial. No impact on long-term mortality
Source: PLoS One. 2019 May 23;14(5):e0216983. doi: 10.1371/journal.pone.0216983 (PMC6532873; doi:10.1371/journal.pone.0216983)
Supplement: S1 Fig — (DOCX) [file pone.0216983.s002.docx]

**S1 Fig. Flow chart indicating process of data enrichment by genealogical search**


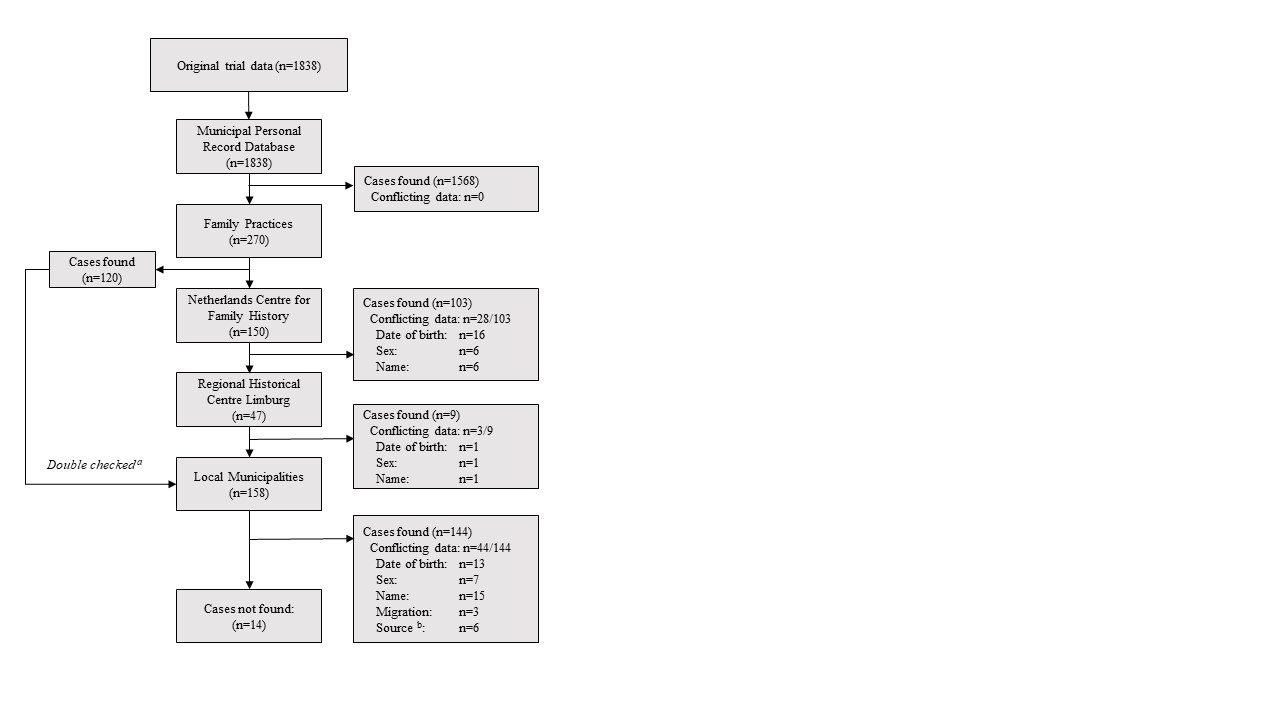


Results from the genealogical search provided input for the definite search as performed by Statistics Netherlands (S2 Fig).

^a^ Registration of personal records by family practices can be considered indicative but not conformational for verity of personal data. Therefore, details provided by family practices were double checked by local municipalities.

^b^ Conflicting source indicates that the used data source shows differences in personal data (e.g. different date of birth or sex) when compared to original trial data (as registered in 1991).
