# Supplementary material for: Influenza vaccination in the elderly: 25 years follow-up of a randomized controlled trial. No impact on long-term mortality
Source: PLoS One. 2019 May 23;14(5):e0216983. doi: 10.1371/journal.pone.0216983 (PMC6532873; doi:10.1371/journal.pone.0216983)
Supplement: S2 Fig — (DOCX) [file pone.0216983.s003.docx]

**S2 Fig. Flow chart indicating definite search process conducted by Statistics Netherlands (CBS), based on data enrichment by genealogical search**


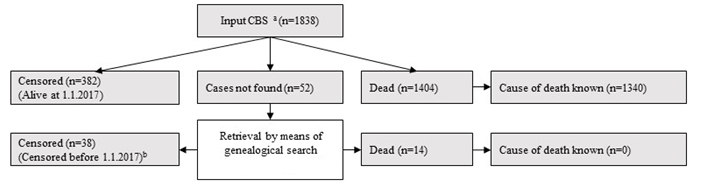


^a^ Input of CBS consisted of the enriched data following the genealogical search(shown in S1 Fig.).

^b^ Of the 38 cases censored before January 1, 2017, 22 cases were censored using follow-up information provided by municipalities and 16 cases were censored using last recorded dates during the trial (dates not shown).
