# Supplementary material for: Influenza vaccination in the elderly: 25 years follow-up of a randomized controlled trial. No impact on long-term mortality
Source: PLoS One. 2019 May 23;14(5):e0216983. doi: 10.1371/journal.pone.0216983 (PMC6532873; doi:10.1371/journal.pone.0216983)

**S3 Fig. Cumulative incidence plot indicating the incidence of pulmonary related seasonal deaths in participants with lung disease (n=244)**


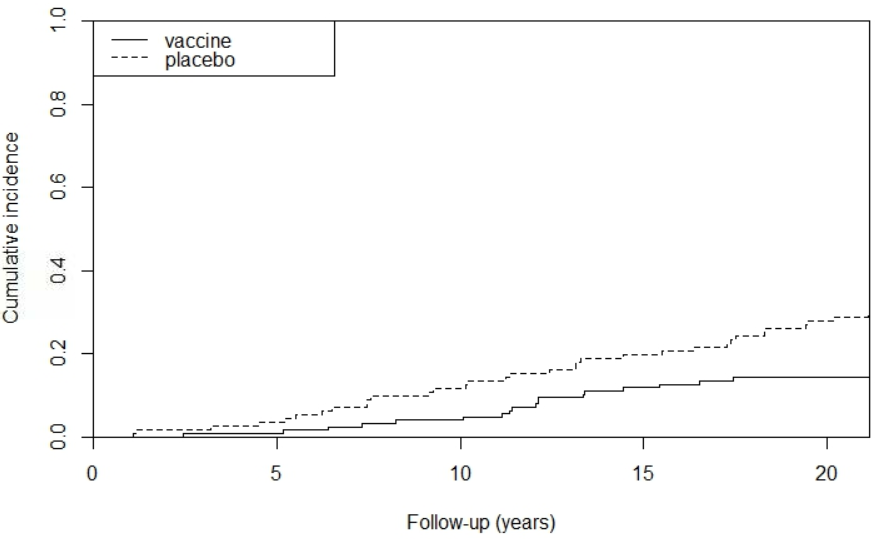

Supplement: S3 Fig — (DOCX) [file pone.0216983.s004.docx]
