# Supplementary material for: Influenza vaccination in the elderly: 25 years follow-up of a randomized controlled trial. No impact on long-term mortality
Source: PLoS One. 2019 May 23;14(5):e0216983. doi: 10.1371/journal.pone.0216983 (PMC6532873; doi:10.1371/journal.pone.0216983)
Supplement: S5 Fig — (DOCX) [file pone.0216983.s006.docx]

**S5 Fig. Crude Kaplan-Meier survival curves for vaccinated and unvaccinated diabetic patients (n=59)**


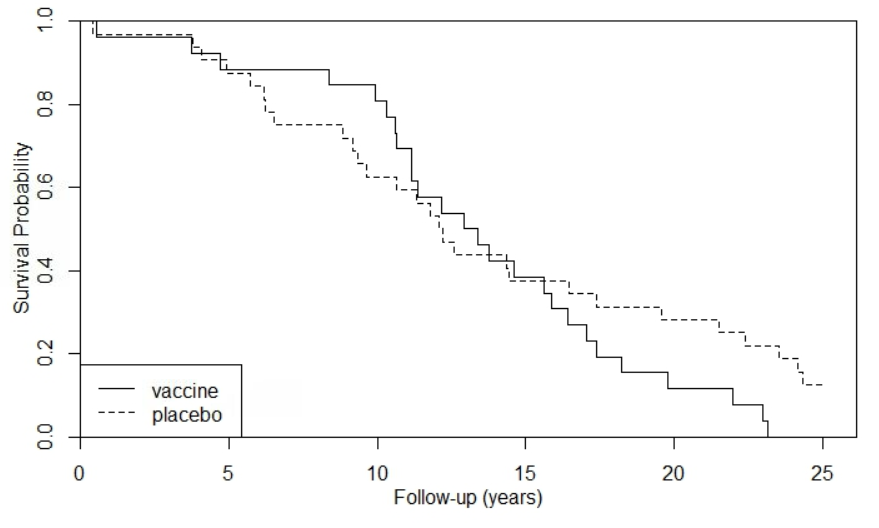
An inverse relation between vaccination and survival after approximately 15 years of follow-up is shown.
